# UNDERSTANDING AND ADDRESSING IMMUNOSENESCENCE

**DOI:** 10.1093/geroni/igz038.774

**Published:** 2019-11-08

**Authors:** Robbert Van Der Most

**Affiliations:** GSK Vaccines, Rixensart, Belgium

## Abstract

Aging comes with an increased impact of infectious disease in terms of hospitalization, morbidity and mortality. This increased susceptibility to infection appears to be linked to age-related changes in the immune system and its capacity to respond to infection and vaccination. Importantly, this phenomenon occurs despite existence of pre-existing immune memory. The age-related weakening of the immune response is referred to as “immunosenescence”. Immunosenescence operates at several levels of the immune system and is multifactorial. Recent advances in systems immunology have shed new light on the immunological processes that may drive the age-related changes in immune response to infection and vaccination. However, gaps in our understanding still exist at basic and translational research levels. One approach to counteract this is the development and implementation of innovative vaccines against the pathogens with particular risks for older adults. The use of innovative immune adjuvants holds promise for the development of such vaccines.

